# Cohort profile: the Saskatchewan Rural Health Study—adult component

**DOI:** 10.1186/s13104-017-3047-1

**Published:** 2017-12-11

**Authors:** Punam Pahwa, Masud Rana, William Pickett, Chandima P. Karunanayake, Khalid Amin, Donna Rennie, Josh Lawson, Shelley Kirychuk, Bonnie Janzen, Niels Koehncke, James Dosman

**Affiliations:** 1 Canadian Centre for Health and Safety in Agriculture, University of Saskatchewan, 104 Clinic Place, PO Box 23, Saskatoon, SK S7N 2Z4 Canada; 2 Department of Community Health and Epidemiology, University of Saskatchewan, Health Science Building, 107 Wiggins Road, Saskatoon, SK S7N 5E5 Canada; 3 Department of Public Health Sciences, Queen’s University, Carruthers Hall, Kingston, ON K7L 3N6 Canada; 4 College of Nursing, University of Saskatchewan, 104 Clinic Place, Saskatoon, SK S7N 2Z4 Canada

**Keywords:** Rural, Small town, Respiratory, Asthma, Chronic bronchitis, Longitudinal, Farming, Non-farming

## Abstract

**Objectives:**

Less is known about the respiratory health of general farming and non-framing populations. A longitudinal Saskatchewan Rural Health Study (SRHS) was conducted to explore the association between *individual* and *contextual factors* with respiratory health *outcomes* in these populations. Hence, the objectives are to: (i) describe the updated methodology of longitudinal SRHS—an extension of baseline survey methodology published earlier; (ii) compare baseline characteristics and the prevalences of respiratory health outcomes between drops-outs and completers; and (iii) summarize key findings based on baseline survey data.

**Results:**

The SRHS was a prospective cohort study conducted in two phases: baseline survey in 2010 and a follow-up in 2014. Each survey consisted of two components, self-administered questionnaire and clinical assessments. At baseline, 8261 participants (≥ 18 years) (4624 households) and at follow-up, 4867 participants (2797 households) completed the questionnaires. Clinical assessments on lung functions and/or allergies were conducted among a sub-group of participants from both the surveys. To date, we published 15 peer-reviewed manuscripts and 40 abstracts in conference proceedings. Findings from the study will improve the knowledge of respiratory disease etiology and assist in the development and targeting of prevention programs for rural populations in Saskatchewan, Canada.

**Electronic supplementary material:**

The online version of this article (10.1186/s13104-017-3047-1) contains supplementary material, which is available to authorized users.

## Introduction

The longitudinal Saskatchewan Rural Health Study (SRHS) was conducted in 2010 and 2014 in the province of Saskatchewan, Canada. Its primary aim was to explore the hypothesis that *individual* (cigarette smoke, obesity) and *contextual factors* (socio-economic, access to health services), are associated with respiratory *outcomes* of asthma, chronic bronchitis, chronic obstructive pulmonary disease and lung function, after controlling for principal covariates (age, gender). Evidence about adverse respiratory effects in agricultural populations including asthma, reductions in lung function, acute inflammatory responses, and other respiratory symptoms has mainly been derived from studies of swine and poultry workers [1–16]. Studies of grain elevator workers have also demonstrated similar detrimental respiratory effects [17–20]. Although inferences may be drawn from these selected worker populations, less is known about the respiratory status of more general farming and non-farming populations [21].

In response to these gaps in knowledge, we had the opportunity to conduct a longitudinal, population-based study to assess possible determinants of respiratory health among rural farming and non-farming people in the province of Saskatchewan. The SRHS [21] was designed using population health framework (PHF) to address observed gaps in the literature regarding respiratory health of farm and non-farm populations [22]. This theory guided the systematic exploration of how individual and contextual risk factors influence respiratory health outcomes in such contexts. The overall purpose of the SRHS was to examine rural environments, defined broadly, as determinants of respiratory health outcomes in rural people. It had two core objectives: to estimate the strengths of relationships between various determinants and respiratory health outcomes in farmers and small town dwellers, and to conduct a prospective cohort evaluation of respiratory health outcomes in farmers and small town dwellers. However, the objective of this report is to: (i) describe the updated methodology of longitudinal SRHS—an extension of baseline survey methodology published earlier; (ii) compare baseline characteristics and the prevalences of respiratory health outcomes between drops-outs and completers; and (iii) summarize key findings based on baseline survey data.

## Main text

The SRHS involved a prospective cohort conducted in two phases: the baseline survey and a 4-year follow-up survey. Prior to the commencement of baseline survey, a pilot study was conducted to optimize the content and administration of the baseline questionnaire [23]. Pilot project responses guided us to modify several questions in the baseline survey questionnaire.

The rural municipal and small town councils provided the taxation lists to the project manager, which were used to compile a registry of mailing addresses [21]. We sampled a population of 11,982 tax paying households to create a database of study population based on sample size calculations and assuming 30% response rate [24]. To maximize the response rates for both baseline and follow-up surveys, we adopted a modified version of the Dillman total design method for mail and telephone surveys [25] in the administration of questionnaires. Dillman’s method comprises of a series of mail contacts with the prospective study participants. In both surveys: (i) study packages contained a letter of invitation, an information pamphlet, and the questionnaire so that recruitment and data collection occurred simultaneously; (ii) these study packages were addressed personally and sent via first class mail to all households; and (iii) a key informant in each household was asked to provide household level information and then to complete a section for each adult living in the household.

### Questionnaire development

Our study and questionnaire instrument were based on the theoretical framework of Health Canada’s Population Health Framework. It’s mentioned in our earlier manuscript [21] that “A panel consisting of the SRHS research team and two community members (one from a RM and one from a small town) developed the study questionnaire. The questionnaire was designed to include key measures required to test the population health framework (PHF)” [21]. Some questions were modified in context of the rural populations. Questions in the instruments (household and individual questionnaires) were adopted from our previous work with rural populations, and from other researchers’ questionnaires (for example income question and access to health care related questions from Statistics Canada survey questionnaires; excessive daytime questions from Epworth Sleepiness Scale questionnaire; and Occupational history related questions from the Cross-Canada study of pesticides and health etc.)

### Study design for adult baseline survey

For the baseline survey the study design is explained in detail in our earlier publication entitled ‘The Saskatchewan Rural Health Study: an application of a population health framework to understand respiratory health outcomes’ [21]. Briefly, SRHS baseline component consisted of three stages, which consisted of: recruitment of populations in rural municipalities (RMs) and small towns (stage 1); self-administration of mailed out household and individual questionnaires to the target populations in order to assess contextual and individual factors listed in Additional file 1: Table S1 (stage 2); and obtaining clinical assessments (anthropometric measures, lung function measurements, and allergy testing-see Additional file 1: Table S1) on a sub-population that completed the questionnaires (stage 3).

### Study design for adult follow-up survey

Those who participated in the baseline were followed-up after 4 years and data on individual and contextual factors listed in Additional file 1: Table S1 were collected via mailed-out self-administered questionnaires. Clinical assessments (see Additional file 1: Table S1) were obtained by contacting all those participants who participated in the clinical component of the baseline survey. In order to maintain a high retention rate for the follow-up study, in the interim we remained in touch with the study participants via regular local newsletters/newspapers, and presented results at local council meetings, and other communications with the rural media.

### Clinical assessments at baseline and follow-up surveys

Those who responded positively to the final question (‘would you be willing to be contacted about having breathing and/or allergy tests at a nearby location?’) on the baseline questionnaire were sent a letter of invitation to participate in a clinical assessment. Those who participated in the baseline clinical assessments were contacted and followed-up after 4 years. At both surveys, clinical measurements included the measurement of height, weight, blood pressure, forced expired volume in one second (FEV_1_), forced vital capacity (FVC), FEV_1_/FVC ratio, and maximum mid-expiratory flow rate (FEF_25–75_) and allergy skin prick tests for six allergens. Sensormedics (Anaheim, CA) dry rolling seal spirometers were used for pulmonary function testing [5, 8] and measurements were taken according to standards of the American Thoracic Society criteria [26]. The protocol used to obtain these measurements is described in detail elsewhere [21].

### Study populations

The study populations consisted of the Farm Cohort and the Small Town Cohort recruited from the four quadrants [Northwest (NW), Northeast (NE), Southwest (SW), and Southeast (SE)] of Saskatchewan [21]. Thirty-two rural municipalities (9 from the NW, 8 from each of the NE and SW, and 7 from SE) and 15 small towns (6 from the NW, 2 from the NE, 4 from SW, and 3 from SE) participated in the phase 1—baseline survey. Questionnaires were mailed to 11,004 households in 2010 [21]. Phase 2—follow-up survey was conducted in 2014 and consisted of mailed questionnaires and clinical assessments of individuals who participated in the phase 1—baseline survey. In the follow-up survey, an initial mailing was administered to 4624 households, of which 4454 were deemed eligible (170 letters were returned to the sender).

### Response rates for baseline and follow-up surveys

**Fig. 1:**
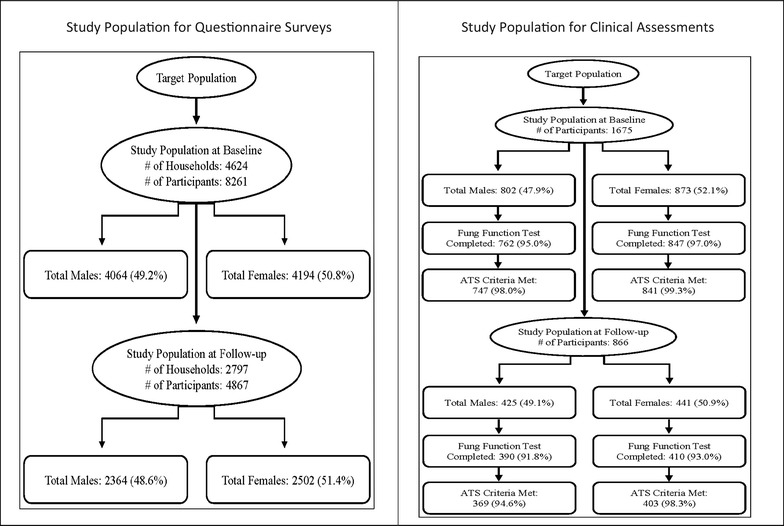
Saskatchewan Rural Health Study: derivation of sample for questionnaire and clinical assessments. Study population for questionnaire and clinical assessments

The response rates for both surveys are provided in Table 1. The response rate of baseline mail-out questionnaire survey was 42%. We obtained completed questionnaires from 4624 households including information about 8261 individuals, 18 years and older (see Fig. 1). In the follow-up survey, questionnaires were returned from 2797 households comprised of 4867 individuals. Of these, 4741 individuals had participated at both time points. There were 126 new individuals who did not participate in the baseline survey but were included at follow-up (see Fig. 1).

**Table 1:** Response rates for the Saskatchewan Rural Health Study in the 2010 baseline and 2014 follow-up surveys

	Baseline (2010)		Follow-up (2014)	
	Small town (n = 15)	RM (n = 32)	Small town (n = 15)	RM (n = 32)
Household addresses (ratepayers) baseline: n = 11,004 Follow-up: n = 4454	5318	5683	2124	2330
Household returned surveys, n (%)	2800 (52.7)	2910 (51.2)	1487 (70.0)	1662 (71.3)
No response, n (%)	2518 (47.3)	2773 (48.8)	637 (30.0)	668 (28.7)
Response rate (based on household addresses) n (%) Baseline: n = 4624^a^ Follow-up: n = 2797	2242 (42.2)	2380 (41.9)	1279 (60.2)	1518 (65.1)
Persons participating Baseline: n = 8261^b^ Follow-up: n = 4741	3785	4472	2050	2691
Age (mean + SE)	56.3 + 0.28	55.9 + 0.22	61.4 ± 0.3	61.0 ± 0.3
Male:female ratio	1774/2007	2292/2179	930/1120	1375/1316
Clinical assessments Baseline: n = 1675 Follow-up: n = 885				
Lung function, n (%)	679 (40.4)	930 (55.3)	356 (40.2)	460 (52.0)
Allergy test, n (%)	686 (40.8)	929 (55.3)	–	–
Both, n (%)	653 (38.8)	896 (53.3)	–	–

^a^Two households were not identified in baseline

^b^Four individuals were not identified by town/RM

–, data not collected

### Response rates for clinical assessments

At baseline, 1675 individuals (802 males and 873 females) gave consent to participate in the clinical assessment component. Of these, 1609 (762 males and 847 females) completed the lung function testing and 1565 (738 males and 827 females) lung function tests met the ATS criteria [26]. Individuals who gave consent to participate in the clinical assessment component at baseline were contacted in 2014 asking their willingness to participate in the clinical component again. Eight hundred sixty-six individuals agreed to participate in lung function testing at the follow-up survey. Of these, 800 (390 males and 410 females) completed the lung function testing and 772 (369 males and 403 females) lung function tests met the ATS criteria (see Fig. 1).

## Comparison of completers vs. drop-outs

There were 4741 people who participated in both surveys (completers) and 3520 who did not participate in the follow-up survey (drop-outs). For the clinical component, 800 of the respondents completed the clinical testing at baseline and follow-up survey and 809 drop-outs did not participate in clinical testing in the follow-up survey. A comparison of baseline characteristics of completers and drop-outs is presented in Table 2.

**Table 2:** Comparison of baseline characteristics between completers (who participated in both surveys) vs. drop-outs (who participated only in the baseline survey)

	Completers (n = 4741)	Drop-outs (n = 3520)	*p* value
Quadrant^a^			0.23
Southwest	854 (18.0)	684 (19.5)	
Southeast	1016 (21.4)	776 (22.1)	
Northeast	1391 (29.3)	1009 (28.7)	
Northwest	1480 (31.2)	1047 (29.8)	
Rural municipality^a^, n (%)			0.0001
Town	2050 (43.2)	1735 (49.4)	
RM	2691 (56.8)	1781 (50.7)	
Location of home^a^, n (%)			0.0001
Farm	2127 (45.1)	1318 (37.8)	
Non-farm	2593 (54.9)	2170 (62.2)	
Sex, n (%)			0.21
Male	2305 (48.6)	1759 (50.0)	
Female	2436 (51.4)	1758 (50.0)	
Age, n (%)			0.0001
18–45	893 (18.8)	1051 (29.9)	
46–55	1261 (26.6)	787 (22.4)	
56–65	1273 (26.9)	675 (19.2)	
> 65	1314 (27.7)	1004 (28.6)	
Mean ± S.E.	57.0 ± 0.2	55.4 ± 0.5	0.002
Socio-economic status			
Income, n (%)			0.0001
Money left at end of month^a^			
Some money	2653 (61.7)	1774 (56.4)	
Just enough money	900 (20.9)	695 (22.1)	
Not enough money	749 (17.4)	679 (21.6)	
Lowest income	134 (3.3)	190 (6.5)	0.0001
Lowest middle income	624 (15.4)	592 (20.3)	
Upper middle income	1437 (35.3)	868 (29.8)	
Highest income	1871 (46.0)	1261 (43.3)	
Education^a^, n (%)			0.0001
≤ grade 12	2716 (57.9)	2225 (64.2)	
> grade 12	1979 (42.2)	1239 (35.8)	
Respiratory health outcomes, n (%)			
Cough^a^	677 (14.4)	558 (16.1)	0.04
Wheeze	1886 (39.8)	1471 (41.8)	0.07
Asthma	386 (8.1)	329 (9.4)	0.05
Chronic bronchitis^a^	281 (6.0)	202 (5.9)	0.82
COPD^a^	91 (1.9)	99 (2.9)	0.006
Lung function (mean ± S.E.)			
Male	N = 577	N = 185	
FVC	4.73 ± 0.04	4.67 ± 0.07	0.44
FEV_1_	3.59 ± 0.03	3.53 ± 0.06	0.42
FEV_1_/FVC*100	75.81 ± 0.31	75.36 ± 0.6	0.49
FEF_25–75_	3.13 ± 0.05	3.07 ± 0.09	0.53
Female	N = 644	N = 203	
FVC	3.40 ± 0.03	3.43 ± 0.05	0.55
FEV_1_	2.66 ± 0.02	2.67 ± 0.04	0.75
FEV_1_/FVC*100	78.02 ± 0.2	77.6 ± 0.5	0.44
FEF_25–75_	2.52 ± 0.04	2.52 ± 0.07	0.99

p values are reported from Chi square test (categorical variable) and t-test (continuous variable)

^a^Total are not adding up to n because there are some missing values

Drop-outs were most likely to have the following characteristics relative to responders: town dwellers, lower socio-economic status in terms of income and education, and higher reported diagnoses of cough, wheeze, asthma, or COPD. No statistically significant differences for lung function values were observed between the two groups.

### Main findings from baseline survey data

Major findings included associations of rural environments with chronic bronchitis, asthma, and decreased lung function. We also collected information on other chronic conditions (secondary outcomes) such as diabetes, mental health, excessive daytime sleepiness etc. We are currently analyzing longitudinal data collected at two time points. Important findings from the pilot study [23, 27] and baseline survey data [21, 28–39] are summarized in Additional file 2: Table S2.

Results based on baseline data have been presented at local, national and international scientific conferences. Forty abstracts have been published in conference proceedings. Two publications from the pilot study and 13 from the baseline survey have been published in peer-reviewed journals. Objectives and important finding of these manuscripts are summarized in Additional file 2: Table S2. Baseline survey data resulted in two MSc theses [40, 41] based on the adult data. Analyses of longitudinal data are ongoing.

## Discussion

To our knowledge, the SRHS is the largest community based study of health in rural populations that has ever been conducted in Canada. We have successfully used Health Canada’s PHF [22] in other population-based epidemiological studies conducted by our research team. The PHF has been used in other two longitudinal studies: (i) the Saskatchewan Farm Injury Cohort Study [24] and (ii) the first nations lung health project [42]. The findings of baseline survey have provided important information about respiratory disease etiology. We have established a baseline for the prevalence of CB and asthma among rural farm and non-farm residents in Saskatchewan against which future developments in control of this disease can be measured. We can use this knowledge of respiratory disease etiology for the development and targeting of prevention and intervention programs for rural population of Saskatchewan.

## Limitations

Strengths and limitations of the SRHS were mentioned in our methodology paper based on baseline survey [21]. There are weaknesses associated with the SRHS and its longitudinal design. As is the case for most designs of this type, it was expensive, time-consuming, and had challenges associated with missing-data due to attrition [43]. Complex methods will be required to analyze longitudinal data that would account for two-layers of complexities: within-household correlation (multiple individuals from the same household) and within-subject correlation (due to repeated observations) and these will be accounted for by using generalized estimating equations (GEE) [43] and robust variance estimation approaches. Additional complications due to missing data in longitudinal studies can be handled via GEE only if missing data are missing completely at random. Several statistical approaches to handle missing data with missing at random or missing not at random mechanisms have been proposed in the recent years [43].

## Additional files

**Additional file 1: Table S1.** Questionnaire and clinical information collected at the baseline and follow-up surveys. Protocol.

**Additional file 2: Table S2.** Objectives and major findings of the manuscripts (based on pilot and baseline studies) published in peer-reviewed journals. Objectives and major findings.

## Abbreviations

ATS: American Thoracic Society
COPD: chronic obstructive pulmonary disease
GEE: generalized estimating equations
GIS: geographic information system
RM: rural municipalities
SEHS: Saskatchewan Rural Health Study
