# Correction to: Cohort profile: the Saskatchewan Rural Health Study—adult component

**DOI:** 10.1186/s13104-018-3294-9

**Published:** 2018-03-26

**Authors:** Punam Pahwa, Masud Rana, William Pickett, Chandima P. Karunanayake, Khalid Amin, Niels Koehncke, Valerie Elliot, Louise Hagel, Josh Lawson, Donna Rennie, Shelley Kirychuk, Bonnie Janzen, Roland Dyck, James Dosman

**Affiliations:** 1 Canadian Centre for Health and Safety in Agriculture, University of Saskatchewan, 104 Clinic Place, PO Box 23, Saskatoon, SK S7N 2Z4 Canada; 2 Department of Community Health and Epidemiology, University of Saskatchewan, Health Science Building, 107 Wiggins Road, Saskatoon, SK S7N 5E5 Canada; 3 Department of Public Health Sciences, Queen’s University, Carruthers Hall, Kingston, ON K7L 3N6 Canada; 4 College of Nursing, University of Saskatchewan, 104 Clinic Place, Saskatoon, SK S7N 2Z4 Canada

## Correction to: BMC Res Notes (2017) 10:732 10.1186/s13104-017-3047-1

Following publication of the original article [1] the authors notified Production that the names of three authors—Valerie Elliot, Louise Hagel, and Roland Dyck—had been unintentionally omitted in the final online version of the manuscript. The corrected author list is shown in this Correction.

The authors also notified Production that the affiliations assigned to William Pickett are incorrect. The correct affiliation is reflected in the author list of this Correction.
