# Supplementary material for: Cohort profile: the Saskatchewan Rural Health Study—adult component
Source: BMC Res Notes. 2017 Dec 11;10:732. doi: 10.1186/s13104-017-3047-1 (PMC5725811; doi:10.1186/s13104-017-3047-1)
Supplement: Supplementary file 1 — Additional file 1: Table S1. Questionnaire and clinical information collected at the baseline and follow-up surveys. Protocol. [file 13104_2017_3047_MOESM1_ESM.docx]

**Table S1. Questionnaire and clinical information collected at the baseline and follow-up**

**surveys**

| **Phase** | **Measurements** |
| --- | --- |
| Baseline 2010  and Follow-up 2014 | **via self-administered questionnaire:**  **Individuals factors:** Family history, Smoking; Alcohol consumption;  Physical activity; Education attainment; Income,  **Contextual factors:** Housing factors; Access to health services (primary  care, emergency medical services, specialist)  **Respiratory health outcomes:** Chronic bronchitis, COPD, Asthma, and Lung function  **Covariates:** Age; Sex; Marital status; and Co-morbidities**:** Diabetes,  Heart Disease, Heart Attack, Hardening of the arteries,  High Blood Pressure, Cystic Fibrosis, Tuberculosis, Stroke, Cancer    **Clinical assessments:** were conducted on a sub-sample who completed the household and individual questionnaires.   - **Clinical measurements:** height, weight , blood pressure, Lung function measurements^*^ (Forced vital capacity, forced expired volume in first second, FEF_25-75_, and FEV_1_/FVC ratio) allergy prick test for six allergens^*^ (alternaria, house dust mite, cladosporium, local grasses, wheat dust, and cat dander) |

** The protocol used to obtain these clinical measurements is explained elsewhere.^23^*
