# Supplementary material for: Cohort profile: the Saskatchewan Rural Health Study—adult component
Source: BMC Res Notes. 2017 Dec 11;10:732. doi: 10.1186/s13104-017-3047-1 (PMC5725811; doi:10.1186/s13104-017-3047-1)
Supplement: Supplementary file 2 — Additional file 2: Table S2. Objectives and major findings of the manuscripts (based on pilot and baseline studies) published in peer-reviewed journals. Objectives and major findings. [file 13104_2017_3047_MOESM2_ESM.docx]

**Table S2. Objectives and major findings of the manuscripts (based on pilot and baseline studies) published in peer-reviewed journals**

| **Reference#** | **Objective** | **Major Findings** |
| --- | --- | --- |
| **Manuscripts Based on Pilot Study** | | |
| **23** | *The objectives of this paper were to identify differences in selecteddemographic characteristics, individual and contextual factors, and variables related to respiratory health between study participants who were willing to participate in subsequent clinical and allergy testing and those who were not willing to participate* | *Based on the pilot project data, we observed that self-selection of study subjects for lung function and allergy tests is driven by the presence of respiratory symptoms (one of the risk factors for low lung function values).* |
| **27** | *To measure the prevalence and determinants of daytime sleepiness measured using the ESS in a rural community population.* | *High levels of sleepiness in this population were common. Obesity was an important risk factor for high ESS score.* |
| **Manuscripts Based on Baseline Survey** | | |
| **21** | **N/A** | **N/A** |
| 28 | *To determine the prevalence of chronic bronchitis (CB) and associated risk factors in farm and nonfarm rural residents in Saskatchewan, Canada.* | *The results suggest that increasing household income and reducing smoking could be primary, modifiable determinants of CB prevalence.* |
| 29 | *The purpose of this research was to carry out a comprehensive study of prevalence, risk factors and co-morbidities of diabetes among adults in rural Saskatchewan and to determine possible differences between those living on and off farms.* | *While known diabetes risk factors are important determinants of diabetes in the agricultural zones of Saskatchewan, on-farm residence is protective and appears related to increased outdoor activities. In contrast, authors have now shown for the first time that exposure to insecticides is an independent risk factor for diabetes among men in rural Canada.* |
| **30** | *To assess the prevalence of excessive daytime sleepiness as measured by the Epworth Sleepiness Scale (ESS) in a rural community population; potential risk factors for OSA were also assessed.* | *High levels of excessive daytime sleepiness in this particular rural population are common and men >55 years of age are at highest risk. Examination of reasons for residual sleepiness and snoring in persons with and without sleep apnea is warranted.* |
| **31** | *To describe the patterning of socioeconomic inequalities in health among rural dwelling women and men in a Canadian province, exploring diversity in associations by measure of socioeconomic position, health outcome, and demographic characteristics.* | *With the exception of occupational skill level, socioeconomic position (SEP) indicators were strongly and inversely related to most health outcomes, often in a graded manner. Associations between SEP and several health outcomes were weaker for older than younger participants (heart attack, high blood pressure, lung disease) and stronger among women compared to men (high blood pressure, lung disease).* |
| **32** | *The objective of this analysis was to examine access to specialized medical care services and several possible determinants of access to services in a distinctly rural population in Canada.* | *The results reveal that accessing*  *specialist care professionals by rural residents was particularly difficult for persons with current respiratory conditions. Furthermore, the magnitude of the risk for encountering difficulty accessing medical specialist care services increased with the greatest distance categories.* |
| **33** | *This report aims to estimate the prevalence of respiratory symptoms and associated environmental risk factors in farming and nonfarming rural-dwelling people.* | *Respiratory symptoms were positively associated with smoking, allergic reaction to inhaled allergens, and other environmental factors for farming and nonfarming populations. The prevalence of respiratory symptoms was higher in the nonfarming rural population compared with the farming rural population. Environmental exposures such as work-related or home environment play an important role in the increased prevalence of respiratory symptoms in farming and non-farming populations.* |
| **34** | *To assess the temporal relationship between farm residency and asthma and hay fever in a Canadian population* | *Farm residency in the first year of life shows a protective effect for adult asthma and hay fever that appears sex specific.* |
| **35** | *This study investigated the relationship between income adequacy, education and obesity in a rural setting.* | *Home location was associated with obesity prevalence in rural Saskatchewan and modified the influence of income adequacy, but not the influence of education, on obesity. Adults not living on farm had an increased risk of obesity and showed a significant impact of income adequacy on obesity.* |
| ***36*** | *The purpose of this study was to assess the rural occupational exposure, including farming, as a determinant of prostate cancer in rural men. The prevalence of prostate cancer and its putative relationship between rural exposures in the Saskatchewan province of Canada were investigated.* | *Workplace exposure to insecticides and fungicides together were statistically significantly associated with prevalence of prostate cancer while farming occupation and duration were not.* |
| ***37*** | *To examine associations between adult atopy, sex, and farm living in a rural Canadian population* | *The prevalence of atopy was 17.8% and was higher in men than women (P < .001). The most common allergy was to grasses (8.8%) followed by house dust mite (HDM) mixed (8.1%). Atopy was significantly lower in those subjects with an early farm exposure and who were female. After adjusting for education, age, and smoking status, both current and early farm exposures were significantly associated with decreased sensitization to cat atopy in women that was stronger with current exposure.* |
| **38** | *The purpose of the present study was to derive prediction equations for commonly used pulmonary function tests in a rural Canadian Caucasian population and to examine whether results of tests were comparable with published equations currently in use ((Crapo, Canadian, and Global Lung Function Initiative 2012)).* | *Although there were slight differences, the prediction equations for rural Caucasians were very much similar to the three published equations, of these two were primarily based on urban subjects (Crapo and Canadian). Therefore, all three published studies can be recommended for the prediction of reference values for rural Canadian Caucasians.* |
| **39** | *The objective the present study was to investigate the association of individual and contextual exposures with lung function by gender in rural-dwelling Canadians.* | *High-risk waist circumference was related to reduced FVC and FEV_1_ for both genders, but the effect was more pronounced in men. Greater pack-years smoking was associated with lower lung function values. Exposure to household smoke was related to reduced FEV_1_, and exposure to livestock, with increased FEV_1_. Lower income adequacy was associated with reduced FVC and FEV_1_.* |
